# Astragalin inhibits autophagy-associated airway epithelial fibrosis

**DOI:** 10.1186/s12931-015-0211-9

**Published:** 2015-04-21

**Authors:** In-Hee Cho, Yean-Jung Choi, Ju-Hyun Gong, Daekeun Shin, Min-Kyung Kang, Young-Hee Kang

**Affiliations:** Department of Food and Nutrition, Hallym University, Chuncheon, Kangwon-do 200-702 Korea

**Keywords:** Astragalin, Autophagy, Epithelial to mesenchymal transition, Oxidative stress, Pulmonary fibrosis

## Abstract

**Background:**

Fibrotic remodeling of airway and lung parenchymal compartments is attributed to pulmonary dysfunction with an involvement of reactive oxygen species (ROS) in chronic lung diseases such as idiopathic pulmonary fibrosis and asthma.

**Methods:**

The *in vitro* study elucidated inhibitory effects of astragalin, kaempferol-3-O-glucoside from leaves of persimmon and green tea seeds, on oxidative stress-induced airway fibrosis. The *in vivo* study explored the demoting effects of astragalin on epithelial to mesenchymal transition in BALB/c mice sensitized with ovalbumin (OVA).

**Results:**

The exposure of 20 μM H_2_O_2_ for 72 h accelerated E-cadherin loss and vimentin induction in airway epithelial BEAS-2B cells, which was reversed by non-toxic astragalin at 1–20 μM. Astragalin allayed the airway tissue levels of ROS and vimentin enhanced by OVA challenge. Collagen type 1 production increased in H_2_O_2_–exposed epithelial cells and collagen fiber deposition was observed in OVA-challenged mouse airways. This study further investigated that the oxidative stress-triggered autophagic regulation was responsible for inducing airway fibrosis. H_2_O_2_ highly enhanced the expression induction of the autophagy-related beclin-1 and light chains 3A/B (LC3A/B) within 4 h and astragalin blocked such induction by H_2_O_2_. This compound deterred the ROS-promoted autophagosome formation in BEAS-2B cells. Consistently, in OVA-sensitized mice the expression of beclin-1 and LC3A/B was highly induced, and oral administration of astragalin suppressed the autophagosome formation with inhibiting the induction of these proteins in OVA-challenged airway subepithelium. Induction of autophagy by spermidine influenced the epithelial induction of E-cadherin and vimentin that was blocked by treating astragalin.

**Conclusion:**

These results demonstrate that astragalin can be effective in allaying ROS-promoted bronchial fibrosis through inhibiting autophagosome formation in airways.

## Introduction

Autophagy, a catabolic process, plays a role in the elimination of damaged organelles and protein aggregates and in the turnover of essential proteins [1,2]. This process is activated by a diverse array of cellular stressors such as endoplasmic reticulum (ER) stress and microbial infection [3,4]. Especially, the autophagic pathways are modulated by oxidative stress response to oxygen tension and oxidant [5]. There is an emerging body of evidence that autophagy regulates various cellular processes and cell fates, including cellular death and senescence, inflammation and immune function [6]. In addition, maladaptive or pathogenic aftereffects such as aberrant accumulation of proteins are observed. Accordingly, impaired autophagy has been implicated in many pathological conditions of neurological disorders, inflammatory bowel disease, diabetes and cancer [7]. Targeting autophagic pathways and their regulatory components may lead to the development of therapeutics.

Some environmental factors such as air pollutants may cause an extreme increase of reactive oxygen species (ROS) generation in the airways [8]. Oxidative stress contributes to airway and lung damage and consequently to several respiratory inflammatory diseases, including cystic fibrosis and chronic obstructive pulmonary disease [9]. Also, excessive production of ROS is thought to play a pivotal role in the pathogenesis of asthma, where exhaled levels of ROS positively correlate with disease severity [10]. Recent investigation has shown a crucial involvement of autophagy in the pathogenic processes underlying pulmonary diseases [11]. Indeed, autophagy is observed in the lung in response to oxidative stress evoked by the exposure to environmental toxicants [7,11]. Thus, the role of autophagy in pulmonary toxicity may offer new clues to strategies to treat lung injury linked to oxidative stress [12]. However, whether autophagy is responsible for promoting cell survival or cytotoxicity in the airways and lung remains elusive. In addition, the function of autophagy in the disease pathogenesis is still unclear and may entail impaired or elevated autophagic activity or imbalances in the activation of autophagic proteins.

Asthma is associated with a cytokine milieu that promotes transforming growth factor-β1 (TGF-β1)-affiliated airway remodeling and loss of lung function [10]. TGF-β1, a pleiotropic cytokine that has been established as a central mediator of tissue fibrosis, regulates autophagy and manipulates many critical aspects of disease-conditions associated with fibrosis and injury responses [13]. Hence, understanding the cellular mechanism of TGF-β1-related autophagic process is crucial for identifying potential new therapeutic targets. On the other hand, a recent report indicates that fibroblasts in idiopathic pulmonary fibrosis acquire resistance to apoptosis and autophagy [14]. Autophagy inhibition induces acceleration of epithelial cell senescence and myofibroblast differentiation of lung fibroblasts which is an underlying mechanism of the pathogenesis of idiopathic pulmonary fibrosis [15].

**Figure 1:**
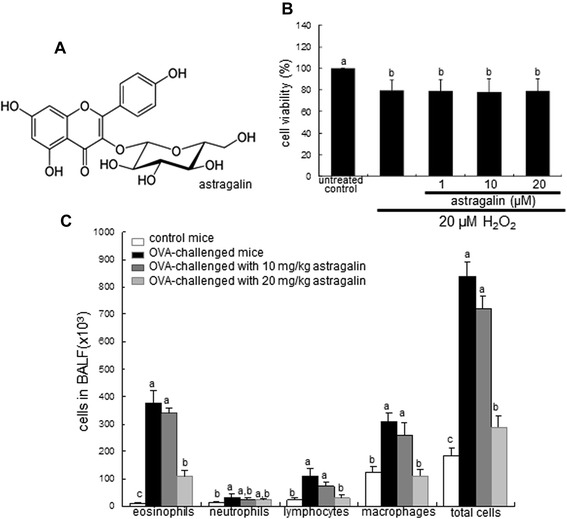
Chemical structure of astragalin **(A)**, cytoprotection of astragalin against H_2_O_2_ **(B)**, and number of neutrophils, eosinophils and basophils in the bronchoalveolar lavage fluid (C, BALF). BEAS-2B cells were cultured with 20 μM H_2_O_2_ in the absence and presence of 1–20 μM astragalin for 72 h **(B)** and cell viability (mean ± SEM, n = 4) was measured by MTT assay. BALF was harvested from BALB/c mice (n = 6, each group) 24 h after the final challenge with OVA or PBS vehicle **(C)**. The bar graphs represent quantitative results and means without a common letter differ, *P* < 0.05.

Astragalin (Figure 1A), kaempferol-3-O-glucoside from leaves of persimmon and green tea seeds, possesses antiinflammatory activity [16,17]. One investigation reveals that pretreatment with astragalin can improve survival during lethal endotoxemia and attenuate inflammatory responses in a murine model of lipopolysaccharide (LPS)-induced acute lung injury [17]. Our recent study showed that astragalin antagonized endotoxin-induced oxidative stress leading to epithelial eosinophilia and apoptosis [18]. However, the inhibitory effects of astragalin on pulmonary fibrosis and autophagy are not yet elucidated. Based on the literature evidence that astragalin possesses antioxidant property and anti-allergic activity [17,18], the current study investigated whether astragalin inhibited epithelial to mesenchymal transition (EMT) leading to pulmonary fibrosis and autophagic activity of H_2_O_2_-exposed airway epithelial BEAS-2B cells and in ovalbumin (OVA)-challenged mice. Furthermore, the interconnection between pulmonary fibrosis and autophagy under oxidative episodes was explored to emphasize a role underlying autophagy in asthmatic fibrosis.

## Materials and Methods

### Chemicals

M199, human epidermal growth factor (EGF), hydrocortisone, gelatin, human insulin, apotransferrin and H_2_O_2_ were obtained from the Sigma-Aldrich Chemical (St. Louis, MO), as were all other reagents, unless specifically stated elsewhere. Fetal bovine serum (FBS), penicillin-streptomycin and trypsin-EDTA were purchased from the Lonza (Walkersville, MD). The human bronchial airway epithelial cell line BEAS-2B cells were provided by the American Type Culture Collection (Manassas, VA). Chicken egg white albumin was purchased from the Sigma-Aldrich Chemical and Imject Alum obtained from the Thermo Fisher Scienctific (Rodkford, IL). Human collagen type-1 antibody was purchased from the Santa Cruz Biotechnology (Santa Cruz, CA). Human E-cadherin antibody was provided by Cell Signaling Technology (Beverly, MA). Antibodies of human beclin-1 and microtubule-associated protein 1 light chain 3A/B (LC3A/B) were obtained from Abcam (Cambridge, UK). Horseradish peroxidase-conjugated goat anti-rabbit IgG, donkey anti-goat IgG and goat anti-mouse IgG were purchased from Jackson Immuno-Research Laboratories (West Grove, PA). Albumin from bovine serum (essentially fatty acid free-BSA) and skim milk were supplied by Becton Dickinson Company (Sparks, MD). 4′,6-Diamidino-2-phenylindole (DAPI) was obtained from Santa Cruz Biotechnology and monodansylcadaverine (MDC) was purchased from Sigma-Aldrich Chemical. Astragalin was dissolved in dimethyl sulfoxide (DMSO) for live culture with cells; a final culture concentration of DMSO was <0.5%.

### BEAS-2B cell culture and viability

The human lung epithelial BEAS-2B cells were cultured in 25 mM HEPES-buffered M199 containing 10% FBS, 2 mM L-glutamine, 100U/ml penicillin, 100 μg/ml streptomycin supplemented with 2.5 μg/ml insulin, 361 ng/ml hydrocortisone, 2.5 μg/ml apotransferrin and 20 ng/ml EGF. BEAS-2B cells were sustained in 90-95% confluence at 37°C in an atmosphere of 5% CO_2_. In order to induce airway fibrosis, BEAS-2B cells were pretreated with 1–20 μM astragalin and then stimulated with 20 μM H_2_O_2_ for up to 72 h. Non-H_2_O_2_-treated cells were also incubated under the same conditions as those used for the H_2_O_2_ protocols. Astragalin did not improve the epithelial injury due to 20 μM H_2_O_2_ (Figure 1B).

### Western blot analysis

Whole BEAS-2B cell lysates were prepared in 1 mM Tris–HCl (pH 6.8) lysis buffer containing 10% SDS, 1% glycerophosphate, 0.1 mM Na_3_VO_4_, 0.5 mM NaF and protease inhibitor cocktail. Cell lysates containing equal amounts of proteins and equal volume of culture media were electrophoresed on 6-15% SDS-PAGE and transferred onto a nitrocellulose membrane. Blocking a non-specific binding was performed using either 3% fatty acid-free BSA or 5% non-fat dry milk for 3 h. The membrane was incubated overnight at 4°C with a specific primary antibody of E-cadherin, vimentin, collagen type-1, beclin-1 and LC3A/B. The membrane was then applied to a secondary antibody conjugated to horseradish peroxidase for 1 h. Following another triple washing, the target protein was determined using the Immobilon Western Chemiluminescent HRP Substrate (Millipore Corp., Billerica, MA) and the Agfa medical X-ray film blue (Agfa HealthCare NV, Mortsel, Belgium). Incubation with mouse anti-human β-actin antibody was conducted for the comparative control.

### Murine asthma models

Six week-old male BALB/c mice (Hallym University Breeding Center for Laboratory Animals) were used in the present study. Mice were kept on a 12 h light/12 h dark cycle at 23 ± 1°C with 50 ± 10% relative humidity under specific pathogen-free conditions, fed a non-purified diet (RodFeed^TM^, DBL, Umsung, Korea) and were provided with water *ad libitum* at the animal facility of Hallym University. The non-purified diet composition is as follows: NLT (Not Less Than) 20.5% crude protein, NLT 3.5% crude fat, NMT (Not More Than) 8.0% crude fiber, NMT 8.0% crude ash, NLT 0.5% calcium and NLT 0.5% phosphorus. Mice were allowed to acclimatize for 1 week before beginning the experiments. Mice were divided into four subgroups (n = 6 for each subgroup). Mice were sensitized with 20 μg OVA dissolved in a solution of 30 μl PBS and 50 μl Imject Alum by a subcutaneous injection twice on day 0 and day 14. For the dietary interventions, 0.1 ml astragalin solution (10 or 20 mg/kg BW) was orally administrated to OVA-sensitized mice 1 h before challenge. On day 28, 29 and 30, 5% OVA inhalation to mice was performed for 20 min in a plastic chamber linked to an ultrasonic nebulizer (Clenny^2^ Aerosol, Medel, Italy). Control mice were sensitized and challenged with PBS as the OVA vehicle. Twenty four hours after the latest provocation (day 30), all mice were killed with an anesthetic (2 μl/kg rompun and 8 μl/kg zoletil, i.p.). The trachea was cannulated, and both lungs and airways were rinsed in 1 ml PBS for the collection of bronchoalveolar lavage fluid (BALF). The numbers of inflammatory cells including eosinophils in BALF were determined using a Hemavet HV950 Multispecies Hematologic Analyzer (Drew Scientific, Oxford, CT). The right lungs were collected, frozen in liquid nitrogen and kept at −80°C until used for Western blotting. Left lungs were preserved and fixed in 4% paraformaldehyde, and then used for immunohistochemical analyses. The total inflammatory cell number in the BALF of mice exposed to OVA increased with a predominance of eosinophils (Figure 1C). The oral administration of astragalin deterred the increase in the number of eosinophils in OVA-treated mice, indicating that astragalin alleviated airway inflammation.

The present study was approved by the Hallym University Institutional Review Board and Committee on Animal Experimentation (hallym R2014-6). All experiments were performed in compliance with the University’s Guidelines for the Care and Use of Laboratory Animals. No mice were dead and no apparent signs of exhaustion were observed during the experimental period.

### Measurement of ROS production

The lung tissue production of ROS was measured using 2′-7′-dichlorofluorescein diacetate (DCF-DA) that is hydrolyzed and oxidized by ROS to a fluorescent compound 2′-7′-DCF. After lysation and incubation of lung tissue extracts with DCF-DA for 30 min, the fluorescence intensity was read in a Fluoroskan reader (Thermo Fisher Scientific, Waltham, MA) with an appropriate filter.

### Immunohistochemical staining

For the immunofluorescent histochemical analysis, paraffin-embedded lung tissue sections (5 μm thickness) were deparaffinized and hydrated. The sections were pre-incubated in a boiling sodium citrate buffer (10 mM sodium citrate, 0.05% Tween 20, pH 6.0) for antigen retrieval. The tissues were blocked with 5% BSA in PBS for 1 h. Specific primary antibody against mouse vimentin, mouse beclin-1, mouse LC3A/B or mouse α-smooth muscle actin (α-SMA) was incubated with the tissue sections overnight. Subsequently, the tissue sections were incubated for 1 h with FITC-conjugated anti-goat IgG or Cy3-conjugated anti-goat IgG. For identification of nuclei, the fluorescent nucleic acid dye DAPI was applied for 10 min. Stained tissues were mounted on slides using fluorescent mounting medium (Vector Laboratories, Burlingame, CA). Images of each slide were taken using an optical microscope Axioimager system (Zeiss, Gottingen, Germany).

### Masson trichrome staining

For the histological analyses, lung specimens obtained at the end of the experiments were fixed in 10% buffered paraformaldehyde. The paraffin-embedded lung specimen were sectioned at 5 μm thickness, deparaffinized and stained with Masson trichrome for the light microscopic visualization of collagen fibers and muscle fibers. The stained tissue sections were examined using an optical microscope system, and five images were taken for each section.

### MDC staining

Labeling of autophagic vacuoles was carried out by using the autofluorescent compound MDC. BEAS-2B cells were treated with 20 μM H_2_O_2_ to in the presence of 1–20 μM astragalin for 4 h on a glass-chamber slide and were incubated in MDC for 30 min. Following the incubation, cells were washed three times with PBS and immediately photographed with a fluorescence microscope.

The formation of autophagosomes in lung tissues was measured by using lung specimen fixed in 10% buffered paraformaldehyde. The paraffin-embedded lung tissue sections were deparaffinized and stained with MDC for the microscopic visualization. The fluorescent DAPI was applied for the nuclear counterstaining.

### Statistical analysis

The results were expressed as mean ± SEM for each treatment group in each experiment. Statistical analyses were performed using Statistical Analysis Systems statistical software package (SAS Institute, Cary, NC). Significance was determined by one-way analysis of variance, followed by Duncan range test for multiple comparisons. Differences were considered significant at *P* < 0.05.

## Results

### Inhibition of airway EMT and fibrosis by astragalin

**Figure 2:**
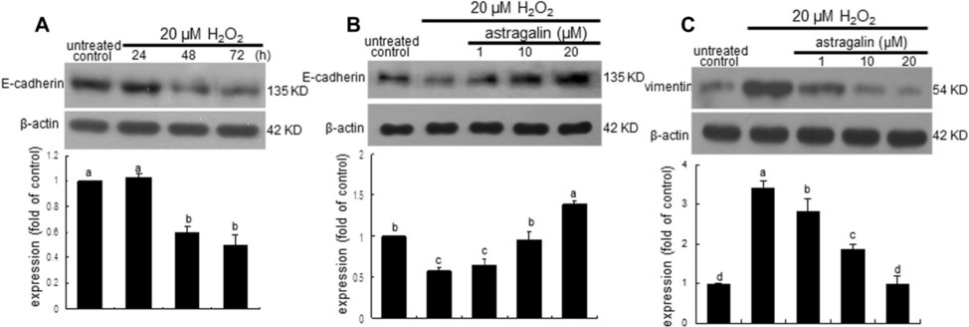
Temporal response of E-cadherin loss **(A)**, and inhibitory effects of astragalin on E-cadherin loss **(B)** and vimentin induction **(C)**. After cells were treated with 1–20 μM astragalin and stimulated with 20 μM H_2_O_2_ for up to 72 h, cell lysates were prepared for Western blotting with a primary antibody against E-cadherin and vimentin. β-Actin protein was used as an internal control. The bar graphs (mean ± SEM, n = 3) represent quantitative results of the upper bands obtained from a densitometer. Means without a common letter differ, *P* < 0.05.

The loss of the epithelial marker E-cadherin and the gain of the mesenchymal marker vimentin are fundamental events in EMT and important changes in the process of fibrosis [19]. BEAS-2B cells exposed to 20 μM H_2_O_2_ up to 72 h induced a change from epithelial phenotype to mesenchymal (Figure 2A). The expression of E-cadherin was reduced at 48 h following the H_2_O_2_ stimulation of BEAS-2B cells. When 1–20 μM astragalin was added to epithelial cells in the presence of H_2_O_2_, the cellular expression of E-cadherin was restored (Figure 2B). On the other hand, the 72 h stimulation of H_2_O_2_ markedly enhanced the epithelial expression of vimentin (Figure 2C). When 1–20 μM astragalin was supplemented to H_2_O_2_-exposed BEAS-2B cells, the vimentin induction was dose-dependently downregulated. Accordingly, oxidative stress may instigate EMT process and fibrosis in airways, which can be disturbed by supplementing astragalin to the epithelium.

**Figure 3:**
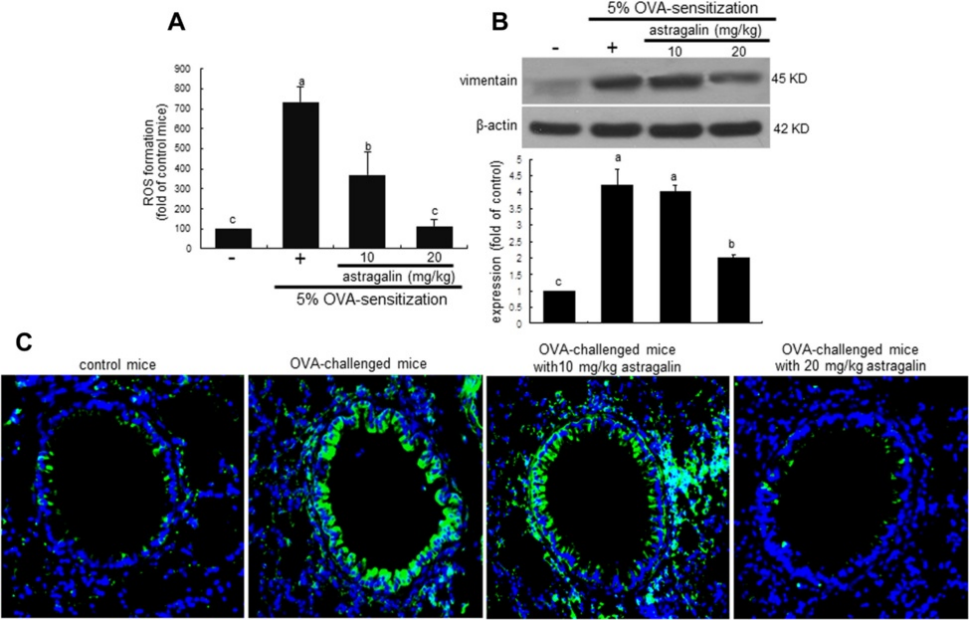
Inhibition of ROS production **(A)** and vimentin induction **(B and C)** by oral administration of 10 mg/kg astragalin in OVA-challenged mouse airway tissues. ROS production was measured by using the fluorescent stain DCF-DA and quantified at λ = 485 nm excitation and λ = 538 nm emission **(A)**. Tissue extracts were subject to Western blot analysis with anti-vimentin **(B)**. The bar graphs (mean ± SEM, n = 3) represent quantitative results of the upper bands obtained from a densitometer. Means without a common letter differ, *P* < 0.05. Vimentin identified as green staining was visualized with a FITC-conjugated secondary antibody and nuclear staining was done with blue dye 4′,6-diamidino-2-phenylindole **(C)**. Each photograph is representative of four mice. Magnification: 200-fold.

This study attempted to determine whether asthmatic injury triggered airway fibrosis, which was dampened by the supplementation of astragalin. The OVA challenge enhanced the ROS production in airway tissues and such elevation was dose-dependently diminished by administrating 10–20 mg/kg astragalin (Figure 3A). Vimentin is commonly used as a marker of cells of mesenchymal origin. Western blot analysis revealed that epithelial expression of vimentin typical in EMT was promoted by the OVA challenge (Figure 3B). However, the oral administration of 20 mg/kg astragalin diminished the elevated induction of vimentin in airway tissues by the OVA episode. In addition, the FITC-green staining of vimentin disappeared in the airway epithelium of 20 mg/kg astragalin-exposed mice (Figure 3C).

**Figure 4:**
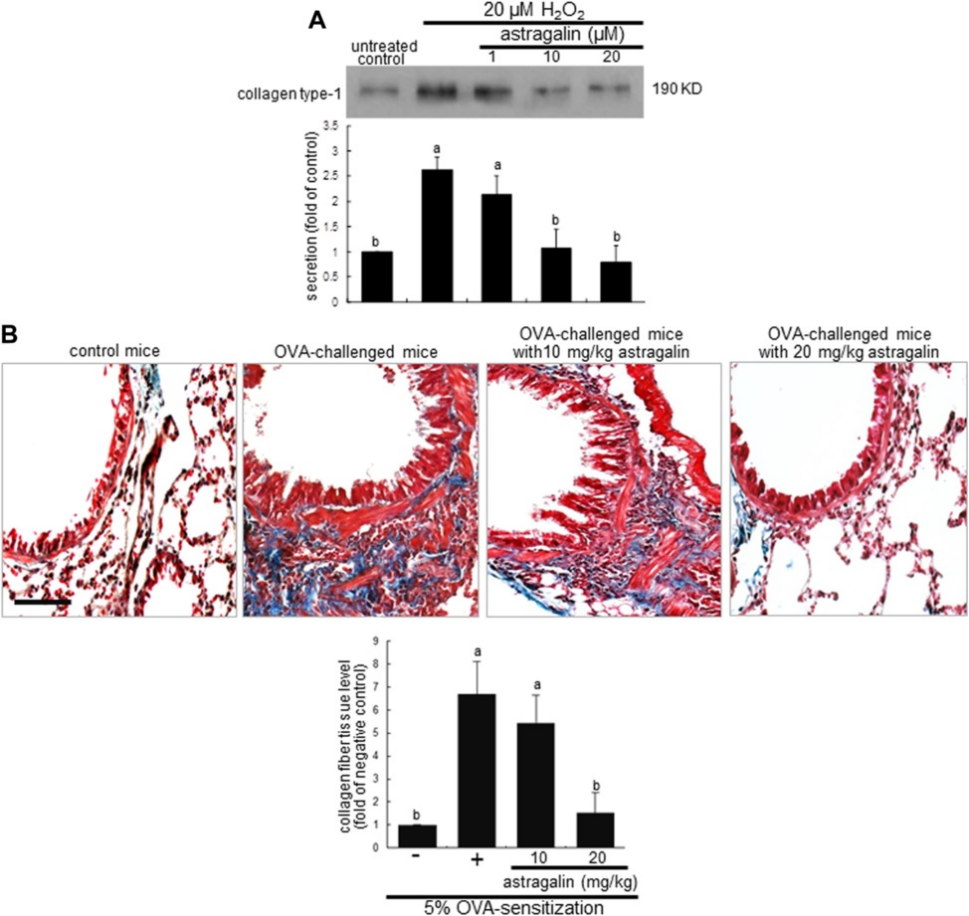
Suppressive effects of astragalin on collagen type-1 production in H_2_O_2_-exposed BEAS-2B cells **(A)** and collagen fiber deposition in OVA-challenged mouse airways **(B)**. BEAS-2B cells were cultured with 20 μM H_2_O_2_ in the absence and presence of 1–20 μM astragalin for 72 h. Culture media were prepared for Western blotting with a primary antibody against collagen type-1 **(A)**. The bar graphs (mean ± SEM, n = 3) represent quantitative results of the upper bands obtained from a densitometer. Means without a common letter differ, *P* < 0.05. Pathological collagen fiber deposition in mouse airways was observed by Masson trichrome staining **(B)**. The collagen fibers were stained in blue and quantified (bottom bar graphs, mean ± SEM). Each photograph is representative of four mice. Bar = 100 μm.

Subepithelial fibrosis in the airways entails the increased deposition extracellular matrix (ECM) such as fibrogenic collagen by myofibroblasts and submucosal resident fibroblasts and fibrocytes expressing profibrotic cytokines [20]. The epithelial production of collagen type-I was elevated in BEAS-2B cells exposed to H_2_O_2_ and such production was suppressed by adding ≥10 μM astragalin (Figure 4A). This study further investigated that astragalin ameliorated the structural remodeling of airways due to increased deposition of ECM components. The collagen fiber deposition was notably observed (blue color) in airway tissues of OVA-challenged mice, evidenced by Masson’s trichrome staining (Figure 4B). There was a dense deposition of collagen fibers around the bronchial tissues of OVA-challenged mice. On the contrary, the oral treatment of OVA-sensitized mice with 10–20 mg/kg astragalin reduced the collagen fiber deposition and alleviated subepithelial fibrosis (Figure 4B). In addition, it should be noted that the OVA sensitization induced epithelial cell modulation and goblet cell hyperplasia (red color).

### Blockade of airway autophagy by astragalin

**Figure 5:**
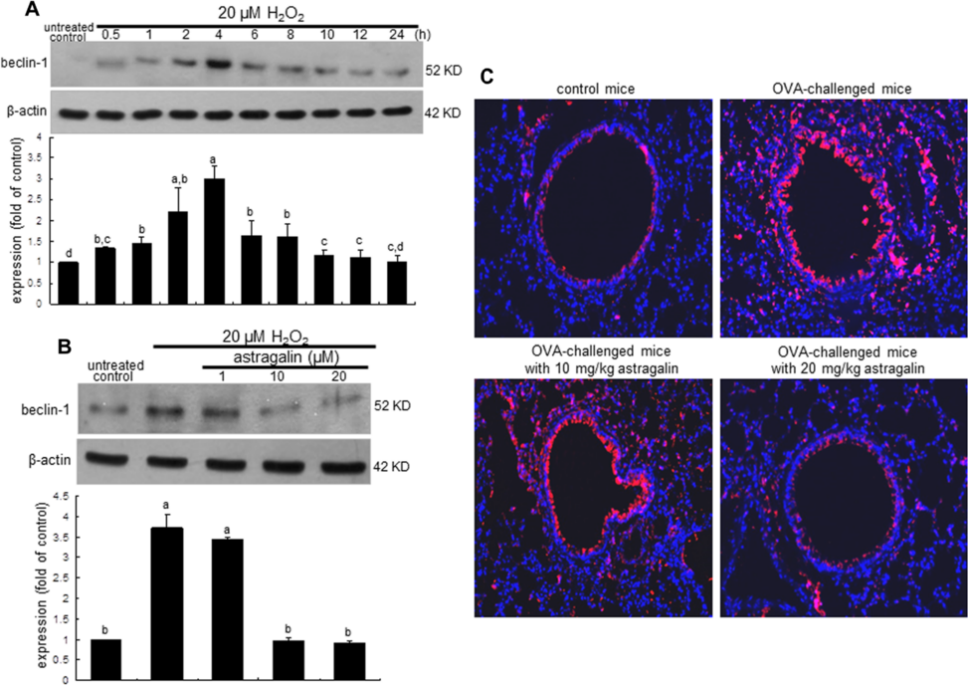
Time course response of beclin-1 expression **(A)**, and inhibition of beclin-1 induction by astragalin in H_2_O_2_-stimulated BEAS-2B cells **(B)** and in OVA-challenged mouse airways **(C)**. BEAS-2B cells were cultured with 20 μM H_2_O_2_ in the absence and presence of 1–20 μM astragalin for up to 24 h. Cell lysates were prepared for Western blotting with a primary antibody against beclin-1 **(A and B)**. β-Actin protein was used as an internal control. The bar graphs (mean ± SEM, n = 3) represent quantitative results of the upper bands obtained from a densitometer. Means without a common letter differ, *P* < 0.05. Immunofluorescence analysis showing inhibition of beclin-1 induction by astragalin in OVA-challenged mouse lung tissues **(C)**. Beclin-1 indentified as red staining was visualized with a Cy3-conjugated secondary antibody and nuclear staining was done with 4′,6-diamidino-2-phenylindole. Each photograph is representative of four mice. Magnification: 200-fold.

**Figure 6:**
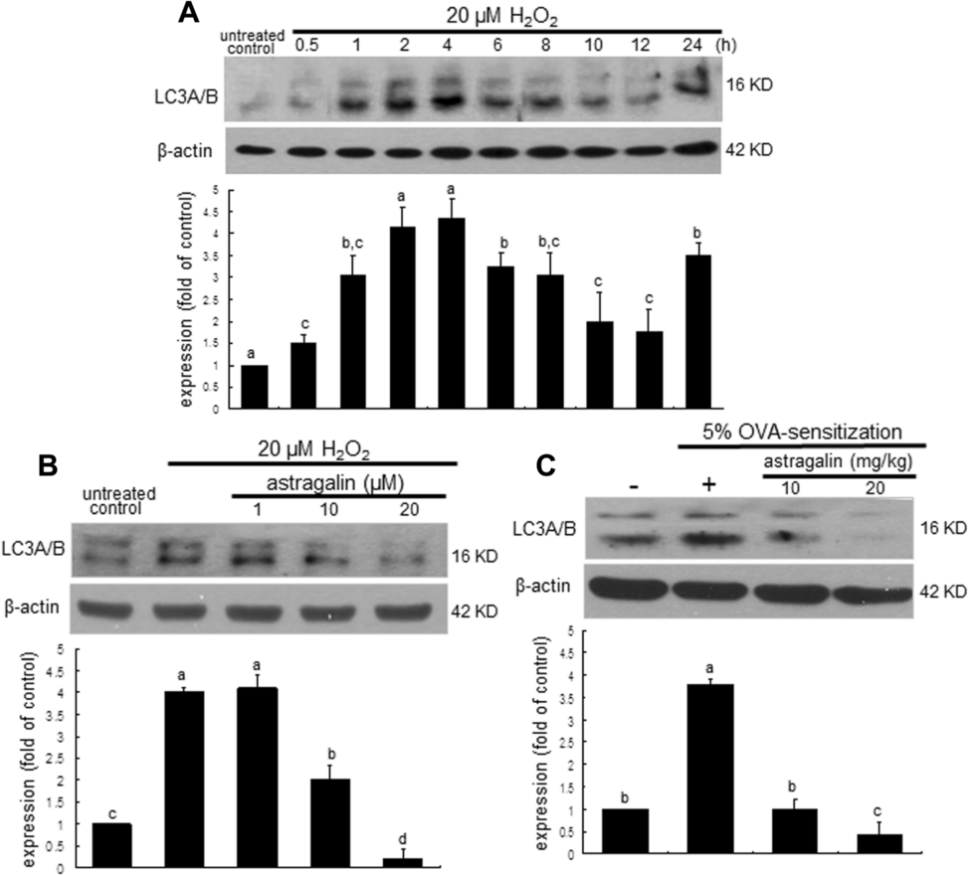
Time course responses of LC3A/B induction **(A)** and its inhibition by astragalin in H_2_O_2_-stimulated BEAS-2B cells **(B)** and in OVA-challenged mouse lung tissues **(C)**. BEAS-2B cells were cultured with 20 μM H_2_O_2_ in the absence and presence of 1–20 μM astragalin for up to 24 h. Cell lysates **(A and B)** and OVA-challenged mouse lung tissue extracts **(C)** were prepared for Western blotting with a primary antibody against LC3A/B. β-Actin protein was used as an internal control. The bar graphs (mean ± SEM, n = 3) represent quantitative results of the upper bands obtained from a densitometer. Means without a common letter differ, *P* < 0.05.

When BEAS-2B cells were incubated with 20 μM H_2_O_2_ up to 24 h, epithelial expression of beclin-1 was strikingly elevated at 4 h after H_2_O_2_ injury and thereafter diminished (Figure 5A). However, its 4 h-epithelial induction was encumbered by treating cells with ≥10 μM astragalin (Figure 5B). Consistently, the OVA inhalation to mice led to the increased levels of the autophagic marker beclin-1 in mouse airway tissues, evidenced by Cy3-red staining (Figure 5C). In contrast, the oral administration of 20 mg/kg astragalin allayed the beclin-1 induction (loss of pinkish staining) in OVA-challenged mouse airways. Microtubule-associated LC3A/B was highly enhanced at 2–4 h after treating 20 μM H_2_O_2_ to BEAS-2B cells (Figure 6A). The epithelial induction of LC3A/B by H_2_O_2_ was diminished in ≥10 μM astragalin-treated BEAS-2B cells exposed to H_2_O_2_ for 4 h (Figure 6B). Similarly, Western blot data showed that the airway tissue levels of LC3A/B increased in OVA-challenged mice, which was dampened by supplementing ≥10 mg/kg astragalin to the mice (Figure 6C).

**Figure 7:**
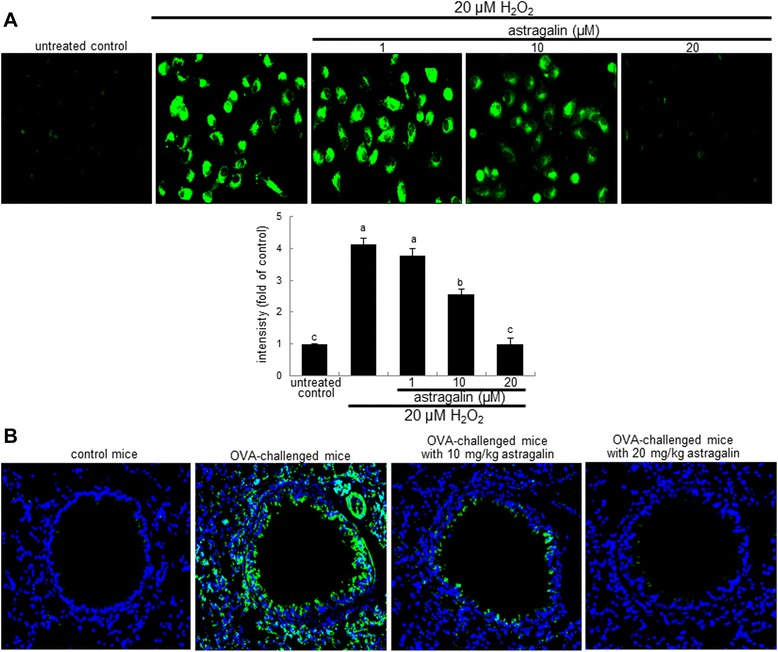
Inhibition of epithelial formation of autophagic vacuoles by astragalin. BEAS-2B cells were cultured with 20 μM H_2_O_2_ in the absence and presence of 1–20 μM astragalin for 4 h and incubated with monodansycadaverine (MDC) at 37°C for 10 min. MDC-loaded cells were visualized by fluorescence microscopy and the fluorescent intensity was quantified **(A)**. The bar graphs (mean ± SEM, n = 3) represent quantitative results of the upper staining. Means without a common letter differ, *P* < 0.05. Immunofluorescence analysis showing inhibition of autophagosome formation by astragalin in OVA-challenged mouse lung tissues **(B)**. Autophagosomes indentified as green staining was visualized with MDC and nuclear staining was done with 4′,6-diamidino-2-phenylindole. Each photograph is representative of four mice. Magnification: 200-fold.

Autophagy is characterized by the formation of double-membrane autophagosomes that fuse with lysosomes to form autolysosomes [21]. This study attempted to confirm whether oxidative stress induced autophagosome formation in BEAS-2B cells. There was a strong MDC staining observed in the cytoplasts of BEAS-2B cells exposed to 20 μM H_2_O_2_ (Figure 7A). When 1–20 μM astragalin was added to the cells, dose-dependent decreases in the number of autophagosomes and in the MDC staining intensity were observed (Figure 7A). Thus, astragalin may be an antagonist to the autophagy induction of BEAS-2B cells in response to H_2_O_2_. Autophagosome formation was further determined in airway tissues of OVA-challenged mice (Figure 7B). There was a strong MDC staining in cells around the airway subepithelium of OVA-inhaled mice. The autophagosomes were formed in similar regions of airways to those of the collagen fiber deposition (Figure 4B). When 10–20 mg/kg astragalin was orally treated to OVA-sensitized mice, the MDC staining was highly diminished (Figure 7B).

### Interconnection between airway fibrosis and autophagy

**Figure 8:**
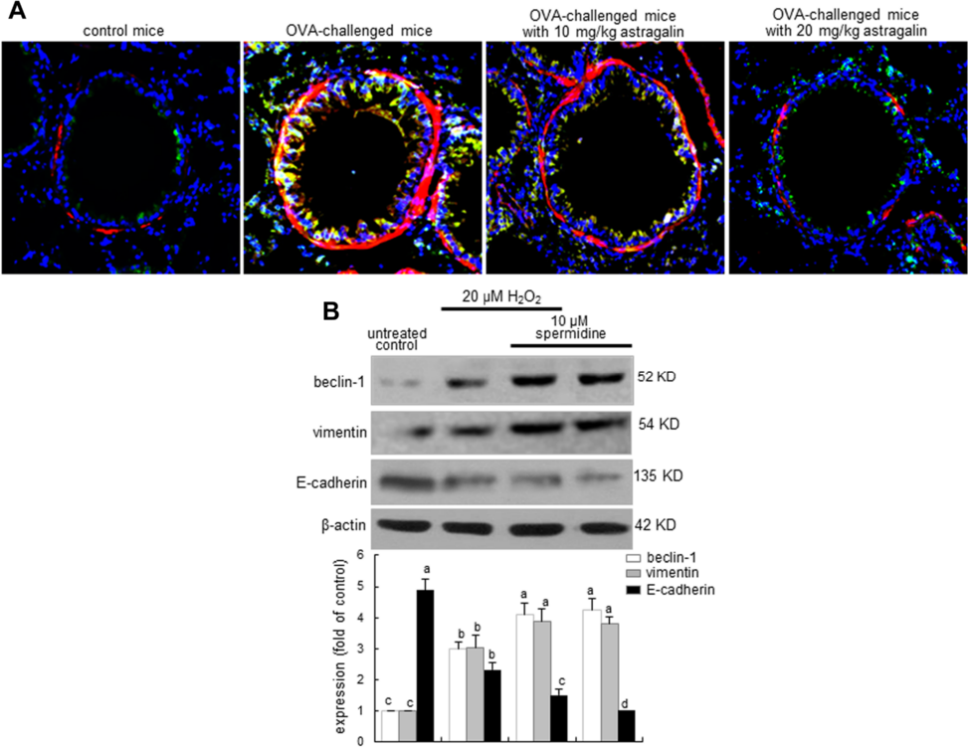
Inhibition of autophagy and airway fibrosis by astragalin in OVA-challenged mouse airways **(A)** and autophagy-induced airway fibrosis in H_2_O_2_-stimulated BEAS-2B cells **(B)**. The induction of α-SMA and LC3A/B was evidenced by immunofluorescence analysis in OVA-challenged mouse lung tissues **(A)**. LC3A/B identified as green staining was visualized with a FITC-conjugated secondary antibody and α-SMA indentified as red staining visualized with a Cy3-conjugated secondary antibody. Nuclear staining was done with 4′,6-diamidino-2-phenylindole. Each photograph is representative of four mice. Magnification: 200-fold. After culturing cells with 20 μM H_2_O_2_ and/or 10 μM spermidine for 72 h, cell lysates were subject to Western bolt analysis with a primary antibody against beclin-1, E-cadherin and vimentin **(B)**. β-actin protein was used as an internal control. The bar graphs (mean ± SEM, n = 3) in the bottom panel represent quantitative results of blots obtained from a densitometer. Means without a common letter differ, *P* < 0.05.

The epithelial induction of both α-SMA and LC3A/B highly increased in airway subepithelium of OVA-challenged mice (Figure 8A). Oral treatment of mice with ≥10 mg/kg astragalin allayed the induction of these proteins by OVA challenge. It should be noted that the induction of these proteins concurrently occurred in similar regions of airway tissues. This study attempted to reveal that epithelial autophagy might be involved in the cellular alteration of airway EMT process and fibrosis. Oxidative stress due to H_2_O_2_ caused the induction of beclin-1 involved in the autophagosome formation (Figures 5A and 8B). In addition, the polyamine spermidine highly increased epithelial beclin-1 induction regardless of H_2_O_2_ presence (Figure 8B). The beclin-1 inducer promoted the vimentin induction and the E-cadherin loss. Accordingly, epithelial autophagy may be responsible for the EMT process in airways leading to fibrosis.

## Discussion

Eight major findings were extracted from this study. 1) Astragalin reduced the number of eosinophils in the BALF of OVA-challenged BALB/c mice, indicating that its administration attenuated airway inflammation entailing eosinophil infiltration in peribronchial and perivascular airway. 2) BEAS-2B cells exposed to 20 μM H_2_O_2_ induced EMT process through reducing epithelial expression of E-cadherin and enhancing epithelial induction of vimentin. 3) When 1–20 μM astragalin was added to epithelial cells in the presence of H_2_O_2_, the cellular expression of E-cadherin was restored, but the vimentin induction was dose-dependently attenuated. 4) Oral administration of astragalin encumbered epithelial cell excrescence and thickening by diminishing the vimentin induction and collagen fiber deposition in OVA-challenged mouse airway. 5) H_2_O_2_ markedly promoted epithelial induction of beclin-1 and LC3A/B within 4 h with increasing the autophagosome formation, which was reversed by astragalin. 6) When ≥10 mg/kg astragalin was treated to OVA-challenged mice, this compound suppressed the induction of beclin-1 and LC3A/B in airway tissues. 7) The protein induction of vimentin, α-SMA and LC3A/B concurrently occurred in similar regions of airway tissues of OVA-inhaled mice. 8) The autophagy inducer promoted the E-cadherin loss and enhanced the vimentin induction, indicating that autophagy appeared to be responsible for the airway EMT. Therefore, astragalin can be effective in deterring airway EMT and fibrosis through modulating oxidative stress-responsive signaling pathway linked to autophagy.

Oxidative stress causes airway and lung damage consequently leading to several respiratory inflammatory diseases such as chronic obstructive pulmonary disease [9,10]. In addition, allergic asthma due to the exposure to environmental antigens may entail an increase in endogenous ROS formation, causing oxidative stress-induced injury to the respiratory system and antioxidant defenses [22,23]. In this study the ROS production was highly induced in airway tissues of OVA-challenged mice. Recent research suggests that the use of redox-based therapy to attenuate levels of ROS provides a potential strategy to alleviate oxidative stress-induced airway inflammation in patients with asthma [22,24]. Therapeutic interventions that reduce the exposure to environmental ROS or augment endogenous antioxidant defenses might be beneficial as adjunctive therapies in asthmatic patients [25]. Our recent study showed that astragalin may be a potent antioxidant antagonizing endotoxin-induced oxidative stress [18]. In addition, astragalin blunted H_2_O_2_ -promoted epithelial apoptosis concomitant with nuclear condensation or caspase-3 activation. Currently, astragalin suppressed the airway infiltration of inflammatory cells following the exposure of mice to the OVA antigen.

Although the specific mechanisms responsible for chronic obstructive pulmonary diseases are still being unraveled, ROS radicals are important mediators of airway tissue damage. Oxidative stress has been implicated as an important molecular mechanism underlying fibrosis in the lungs [26]. However, the causal role of ROS released from inflammatory/interstitial cells in mediating fibrosis is not firmly established. Pulmonary fibrosis, a progressive and lethal lung disease, is characterized by inflammation and accumulation of ECM components [27]. This study showed that the oxidant H_2_O_2_ enhanced the collagen type-1 production and the OVA antigen augmented the collagen fiber deposition in the airway subepithelial compartments. Pulmonary fibrosis as well as how best to relieve an imbalance in ROS production would be major therapeutic challenges for which new strategies are warranted. In the present study astragalin as an antioxidant was effective in encumbering airway fibrosis triggered by oxidative stress. Unfortunately, this study did not examine the key origins of oxidative stress in OVA-induced pulmonary fibrosis. Nevertheless, novel insights into unique therapeutic targets of astragalin for managing ROS-induced pulmonary fibrosis need to be provided.

ROS and markers of oxidative stress play fundamental roles in the pathology of idiopathic pulmonary fibrosis characterized by interstitial fibrosis governing irreversible deformation of pulmonary architecture, possibly through the induction of EMT [28]. One investigation showed that the potent antioxidant N-acetylcysteine inhibited TGF-β1-induced EMT in a rat epithelial cell line and in primary rat alveolar epithelial cells, indicating that oxidative stress might be responsible for pulmonary function and the antioxidant possess its beneficial effects on idiopathic pulmonary fibrosis [29]. In this study astragalin allayed the EMT process by restoring E-cadherin expression and reducing vimentin induction in oxidant-experienced airway epithelial cells and OVA-challenged airway tissues. There is growing evidence suggesting that EMT entails TGF-β1 in alveolar epithelial cells and may contribute to airway remodeling in severe asthma and fibrotic lung diseases [10]. In addition, the association between ROS and TGF-β1-induced fibrosis has been discussed [26]. Oxidative stress elicits the production of cytokines and growth factors that may play a pivotal role in invasive myofibroblastic differentiation and collagen deposition. One can speculate that astragalin alleviated oxidative stress and blocked TGF-β1 production, which might lead to the inhibition of airway EMT and fibrosis.

A recent study shows that TGF-β1 regulates autophagy involved in fibrosis of kidney diseases including tubulointerstitial fibrosis, glomerulosclerosis, and diabetic nephropathy [13]. This study emphasized an emerging role of the autophagy in pulmonary fibrosis. The regulation of epithelial autophagy by TGF-β1 can be implicated in the pathogenesis of asthmatic pulmonary fibrosis. Oxidative stress has been shown to be involved in the pathogenic process of autophagy [7,10,12]. In this study the autophagic stress caused by H_2_O_2_ accelerated the epithelial EMT, and the autophagic process concurrently occurred in airway fibrotic regions caused by OVA inhalation in mice. A recent insight into the cellular and molecular bases of the autophagic process will identify potential new therapeutic targets of pulmonary fibrosis.

Autophagy is required for hepatic fibrogenesis by activated hepatic stellate cells in mice and selective reduction of autophagic activity in fibrogenic cells might be used to treat patients with fibrotic diseases [30]. Consistently, the modulation of autophagy may have the potential to treat common respiratory ailments and disorders. In this study astragalin inhibited the autophagosome formation from oxidant-exposed airway epithelial cells and in airway tissues of OVA-inhaled mice through reducing the induction of beclin-1 and LC3A/B. These findings indicate that oral administration of astragalin may attenuate the oxidative stress-induced autophagy and consequent fibrosis. On the other hand, there are conflicting reports indicating that fibroblasts in idiopathic pulmonary fibrosis are resistant to apoptosis and autophagy concomitant with decreased expression of autophagic beclin-1 [14,31]. The phenolic compounds of rutin and curcumin function as a possible effective stimulator of fatty acid-induced autophagy via mTOR-dependent pathways in nonchemical induced-hepatic stellate cells that can transdifferentiate into myofibroblast-like cells, indicating their benefits for alleviating liver fibrosis [32]. In addition, the autophagy inhibition induces acceleration of epithelial cell senescence [15]. Accordingly, whether autophagy is responsible for promoting epithelial toxicity or survival in airways and lung should be clarified. Ultimately, the unique role of autophagy in pulmonary toxicity linked to oxidative stress may provide novel clues to strategies to treat pulmonary fibrosis [12].

## Conclusion

This study investigated the potential of astragalin as a modulator antagonizing oxidative stress-associated autophagy leading to pulmonary fibrosis. Astragalin restored the expression of the epithelial marker E-cadherin and suppressed the H_2_O_2_ induction of the mesenchymal vimentin in epithelial cells and in OVA-challenged mouse airway tissues. Oral administration of astragalin inhibited subepithelial deposition of collagen fibers in OVA-sensitized mouse airways. In addition, astragalin reduced the bronchial subepithelial induction of the autophagic beclin-1 and LC3A/B enhanced by H_2_O_2_ oxidant and OVA antigen. Ultimately, astragalin encumbered the induction of EMT and fibrosis under the milieu of oxidative stress and autophagic injury in mouse airways. Therefore, astragalin was effective in ameliorating oxidative stress-associated pulmonary fibrosis through disturbing airway EMT and epithelial autophagic stress.
